# *Notes from the Field:* Recurrence of a Multistate Outbreak of *Salmonella* Typhimurium Infections Linked to Contact with Hedgehogs — United States and Canada, 2020

**DOI:** 10.15585/mmwr.mm7032a3

**Published:** 2021-08-13

**Authors:** Michelle A. Waltenburg, Megin Nichols, HaeNa Waechter, Jeffrey Higa, Laura Cronquist, Anne-Marie Lowe, Jennifer K. Adams, Kenai McFadden, Jennifer A. McConnell, Rebecca Blank, Colin Basler

**Affiliations:** ^1^Epidemic Intelligence Service, CDC; ^2^Division of Foodborne, Waterborne, and Environmental Diseases, National Center for Emerging and Zoonotic Infectious Diseases, CDC; ^3^New York City Department of Health and Mental Hygiene, New York; ^4^California Department of Public Health; ^5^North Dakota Department of Health; ^6^Public Health Agency of Canada, Ottawa, Ontario; ^7^Association of Public Health Laboratories, Silver Spring, Maryland; ^8^Oak Ridge Institute for Science and Education, Oak Ridge, Tennessee; ^9^Ventura County Public Health, Oxnard, California.

In July 2020, PulseNet, the national molecular subtyping network for enteric disease surveillance, detected a cluster of 17 *Salmonella* Typhimurium infections. The isolates were closely related genetically to each other (four allele differences) by whole genome sequencing (WGS) analysis and related to isolates from two previous outbreaks of *S.* Typhimurium infections linked to pet hedgehogs (*1*,*2*). An investigation was initiated to characterize illnesses and identify the outbreak source.

A case was defined as the isolation of *S.* Typhimurium closely related by WGS to the outbreak strain in a specimen from a patient with illness onset during April–November 2020. State and local officials interviewed patients about hedgehog exposures and purchase information. Animal and environmental sampling of hedgehog enclosures was conducted at some patient residences. Hedgehog purchase locations were contacted in an attempt to identify a possible common source or supplier of hedgehogs. This activity was reviewed by CDC and was conducted consistent with applicable federal law and CDC policy.*

Forty-nine cases were identified in 25 states, including 14 (29%) in children aged <5 years. Eleven (26%) of 42 patients with available information were hospitalized, and no deaths were reported. Among 36 interviewed patients (or their parents or guardians), 30 (83%) reported hedgehog contact before becoming ill. Seven of 13 patients reported awareness of the risk for *Salmonella* transmission from hedgehogs and other small mammals. Samples collected from hedgehogs in patients’ homes in New York and North Dakota and from a hedgehog habitat in California yielded the outbreak strain of *S.* Typhimurium. Isolates were closely related genetically (23 allele differences). The Public Health Agency of Canada identified 31 cases highly related by WGS to U.S. cases, also linked to hedgehog contact (*3*).

Hedgehog purchase locations were available for 20 of the 36 patients interviewed and included U.S. Department of Agriculture–licensed breeders,^†^ unlicensed breeders, pet stores, and online sales (Figure). No common hedgehog supplier was identified as the source for either the U.S. or Canadian outbreaks. Among 27 identified U.S. hedgehog sources, six breeders were interviewed. All six breeders reported that they provide educational information to new owners when they purchase hedgehogs; four of the six provide information on prevention of disease transmission from pets to humans. Five of the six breeders reported that they work with a veterinarian or veterinary clinic; of these, only one breeder reported having a protocol in place for testing hedgehogs for *Salmonella*.

**FIGURE:**
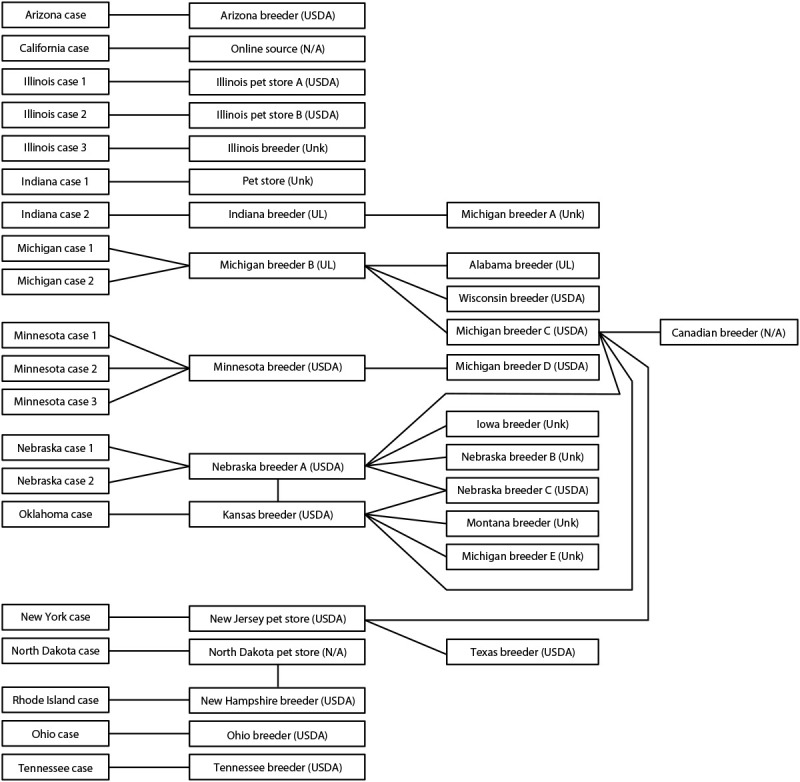
Traceback* of hedgehogs associated with human *Salmonella* Typhimurium infections from patient to hedgehog source (N = 20) — United States, 2020 **Abbreviations:** N/A = license status not applicable; UL = unlicensed; Unk = license status unknown; USDA = U.S. Department of Agriculture licensed. * Traceback for hedgehog distribution from patients back to USDA-licensed hedgehog breeders, unlicensed breeders, pet stores, and online sales. The Michigan, Nebraska, New York, and Oklahoma patients purchased hedgehogs directly from breeders who reported receiving hedgehogs from multiple other breeders. The California patient purchased a hedgehog via an online platform from a seller who had a single hedgehog and did not breed hedgehogs; the source of the seller’s hedgehog is unknown. Names and exact locations of the Illinois breeder, Iowa breeder, Michigan breeder, Montana breeder, Nebraska breeder B, and the pet store associated with Indiana case 1 are unknown. The North Dakota patient obtained the hedgehog during an adoption event at a retail pet store chain; information regarding the source of the hedgehog was obtained from the previous owner who surrendered the hedgehog. USDA licensure status: https://www.aphis.usda.gov/aphis/ourfocus/animalwelfare/ct_awa_regulated_businesses

This particular *Salmonella* strain has continued to cause disease despite targeted outreach to hedgehog breeders and industry groups during two previous outbreaks with the strain linked to hedgehogs (*1*,*2*), highlighting that additional efforts are needed to reduce the prevalence and spread of *Salmonella* among hedgehogs and to limit transmission from hedgehogs to humans. CDC recommendations to pet owners during this outbreak focused on handling hedgehogs safely, including proper hand hygiene (*4*). Recommendations to hedgehog breeders included working with veterinarians experienced in reducing *Salmonella* prevalence in animal populations to evaluate sanitation and husbandry practices and monitoring hedgehogs for *Salmonella* through diagnostic testing.

Prevention and control of *Salmonella* in hedgehogs is complicated because of asymptomatic carriage and persistent or intermittent fecal shedding; however, *Salmonella* mitigation is possible through prevention and control measures focused on good sanitation and husbandry practices (*5*,*6*). To prevent future outbreaks linked to contact with pet hedgehogs, breeders and veterinarians need to educate owners on the risk and prevention of *Salmonella* transmission from hedgehogs and advise that hedgehogs might be inappropriate pets for children aged <5 years. The pet industry, veterinarians, and public and animal health officials could collaborate to help prevent disease transmission to humans by establishing and disseminating information on ways to reduce the prevalence of *Salmonella* in hedgehog breeding colonies intended for use in the pet industry.

## Footnotes

* 45 C.F.R. part 46, 21 C.F.R. part 56; 42 U.S.C. Sect. 241(d); 5 U.S.C. Sect. 552a; 44 U.S.C. Sect. 3501 et seq.

†
https://www.aphis.usda.gov/aphis/ourfocus/animalwelfare/ct_awa_regulated_businesses
